# Glutathione improves low temperature stress tolerance in pusa sheetal cultivar of *Solanum lycopersicum*

**DOI:** 10.1038/s41598-022-16440-8

**Published:** 2022-07-22

**Authors:** Nadia Gul, Parvaiz Ahmad, Tanveer A. Wani, Anshika Tyagi, Saima Aslam

**Affiliations:** 1 Department of Biotechnology, School of Biosciences and Biotechnology, Baba Ghulam Shah Badshah University, Rajouri, 185234 India; 2 Department of Botany and Microbiology, Faculty of Science, King Saud University, Riyadh, 11451 Saudi Arabia; 3 Department of Pharmaceutical Chemistry, College of Pharmacy, King Saud University, Riyadh, 11451 Saudi Arabia; 4 Department of Biotechnology, Yeungnam University, Gyeongsan, Gyeongbuk 38541 Republic of Korea

**Keywords:** Biochemistry, Biotechnology, Physiology, Plant sciences

## Abstract

To investigate the impact of Glutathione (GSH) in mitigating low-temperature stress in Pusa Sheetal cv. of *Solanum lycopersicum* and imparting low-temperature tolerance by evaluating the different physiological responses**.** The plant under research was also being studied for its growth and stress tolerance. Low temperatures (LT) stress was applied to seedlings with or without GSH application 12 h before LT stress (prophylactic dose), after 12 h-LT (preemptive dose), and post 12-h recovery (curative dose). Different concentrations of GSH [0, G1 (0.5 mM), G2 (1 mM) and G3 (2 mM)] against LT stress were used. Antioxidant activities, photosynthesis, growth, and stress tolerance indices were quantified. LT stress caused an oxidative burst in *S. lycopersicum* seedlings of the Pusa Sheetal cv. as indicated by increased peroxidation of lipids and H_2_O_2_ concentration. Glutathione reductase (GR), superoxide dismutase (SOD), catalase (CAT), and ascorbate peroxidase (APX) activities were enhanced. The best concentration was G2 (1 mM), which resulted in a rise in antioxidant activity. Moreover, a decline in lipid peroxidation and H_2_O_2_ levels was also seen. The purpose of this study is to identify the role of GSH in reducing LT stress and to find the best dose concentration. This is the first report to assess the GSH-mediated LT stress tolerance in *S. lycopersicum* (Pusa Sheetal cv.). Therefore, exogenous GSH application of optimal concentration of GSH to LT stressed *S. lycopersicum* can be an effective approach for augmenting the plant detoxification system and promoting its growth and development.

## Introduction

Diverse climatic conditions have led plants more susceptible to different environmental stresses that hinder their survival, development and overall output^1^. Severe temperature fluctuations including low temperature (LT) is among these stresses that have a tremendously deleterious effect on plants. Low temperature alters plant physiology leading to membrane damage, change in lipid composition, chlorosis and different enzyme activity leading to plant necrosis and even death too^2,3^. Change in the fluid state of the membrane is the potential instant response that plant encounters in response to low temperature in addition to the formation of extracellular ice crystals in intercellular spaces if there is a temperature dip below 0 °C^4^. Plants fail to germinate or grow differentially in response to cold stress. LT affects a plant at all stages of development thereby resulting in a decline in crop yield as plants either limit their growth or completely cease it^5^. LT stress alters the basic physiological, anatomical and morphological traits of plants. Photo-inhibition leads to downregulation of photosynthetic activity of plants under LT stress ^6^.

LT acts as a potent inducer of reactive oxygen species (ROS) generation in plants. Plants have mechanisms by which they sequester and scavenge ROS under homeostatic conditions^7^. They have both enzymatic as well as non-enzymatic defense systems against ROS. But in the case of LT stress, the generation of ROS is in excess in many organelles, causing its accumulation thereby disrupting the homeostatic state of plant^8^. One among the non-enzymatic plant antioxidant that plays a characteristic role in the ascorbate glutathione (AsA-GSH) cycle in mitigating ROS is glutathione (GSH). AsA-GSH process controls the levels of H_2_O_2_ in plant cells^9,10^. Plants tend to have a lower GSH/GSSG ratio under stress conditions due to the conversion of GSH to GSSG form upon oxidation^11^. Exogenous supplementation of GSH to plants like mung bean seedlings, strawberries, loquat fruit, ball pepper etc. enhanced their endogenous level of antioxidant content and efficacy of glyoxalase systems to boost their antioxidant capacity under different unfavorable stressful environments^12–15^. However, GSH biosynthesis ceased considerably due to increased H_2_O_2_ and O^2−^ levels in plants like *Sedum alfredii* exposed to Cd stress^16^. The capability of ROS scavenging is an important mechanism to defend against stress associated with abiotic factors and mitigation of chilling injury in vegetables and fruits.

Plants of tropical and sub-tropical areas are cold-sensitive by nature and lack cold mitigating mechanisms. While temperate plants tend to bear the LT stress, various crops like rice, potato, corn, cotton, soyabean and tomato are chilling sensitive plants^17^. One of the most significant fruits is the tomato (*Solanum lycopersicum*) which is a very popular horticultural vegetable crop because of its unique beneficial composition, which makes it anti-oxidant and anti-cancer in nature^18^. But like many other crops, tomatoes too have chilling labile nature that makes it prevail among the category of cold susceptible crop varieties that fail to combat cold stress conditions^17^. Generally, the normal temperature for the fruit set is from 15 to 25 °C, depending upon the variety. Poor anther dehiscence, pollination, and pollen viability all contribute to reduced fruit set at low temperatures. The majority of *S. lycopersicum* cultivars are sensitive to LT at all stages of development, including germination, vegetative growth, and reproduction^19^. However, there is a fair amount of genetic variation within and between tomato species, which could be used to increase tomato cultivars' chilling tolerance. Cold tolerant varieties of *S. lycopersicum* can grow in the greenhouse or field under sub-optimal temperatures due to its productivity benefit^20^. As these tolerant plants grow more vigorously at the initial stages due to more physiological adaptation than the cold-sensitive cultivars. Meanwhile, these have better fruit yield and quality when grown under LT stress regimes. Two cold-set lines of tomato, Balkan and Jemnorrosnij, were crossed and selections were made from the segregating generations of plants that set fruit at up to 8 °C. This resulted in the production and release of the variety Pusa Sheetal^21^. Chilling injury resulting from LT stress is associated with disparity between making and elimination of ROS. Here, we evaluated Chilling injury decrease in pusa sheetal by application of GSH, measuring growth, photosynthetic and antioxidant attributes. The aim of this research was to figure out the importance of GSH in mitigating detrimental effects of cold stress on pusa sheetal cv. of *S. lycopersicum* on oxidative stress, growth and photosynthetic attributes.

An experiment under controlled conditions was carried out to assess the impact of foliar GSH treatment in mediating LT stress in *S. lycopersicum*. Here, we hypothesize that different concentrations of GSH treatment may have the same response under LT stress by regulating the stress response, antioxidant potential and growth. The specific objectives that were evaluated (1) Exogenous application of different concentrations of GSH under LT stress (2) To choose the best concentration based on potential adaption mechanisms involving antioxidant potential, growth and stress markers comparable to that of control. This study lays the foundation for the development of curative measures against LT stress in pusa sheetal cv. of tomato and will help us to frame the different omics-based strategies to combat LT stress.

## Effect of low temperature (LT) and exogenous GSH concentrations on pusa sheetal cv.

### Growth characteristics

**Figure 1:**
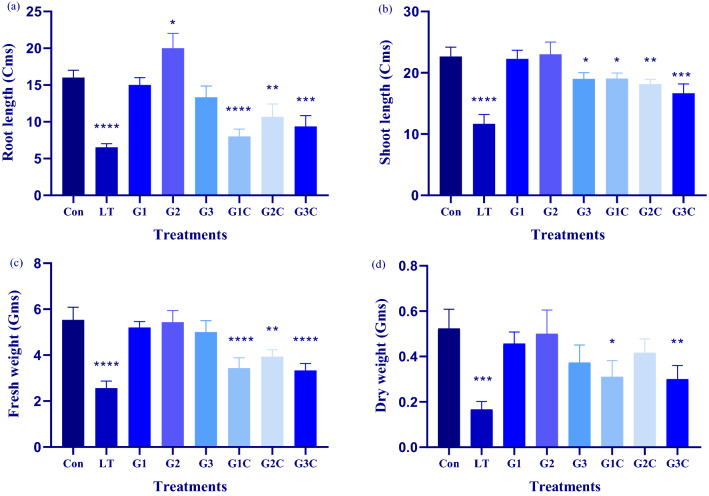
Effect of LT stress and different GSH concentrations on growth characteristics: Forty days after sowing seedlings were used for experimentation pertaining to LT stress and exogenously applied GSH. Post 3 days recovery determination of (**a**) root length (**b**) shoot length (**c**) fresh weight and (**d**) dry weight was done. The data depicts the mean and standard deviation of three replicates. Data followed by (*) determines level of significance (*p* < 0.05) as predicted by Dunnet’s multiple comparison test. Control (Con): 25/18 °C + 0 mM GSH, Low temperature stress (LT): 10/3 °C + 0 mM GSH, G1: 25/18 °C + 0.5 mM GSH, G2: 25/18 °C + 1 mM GSH, G3: 25/18 °C + 2 mM GSH, G1C: 10/3 °C + 0.5 mM GSH, G2C: 10/3 °C + 1 mM GSH, G3C: 10/3 °C + 2 mM GSH.

LT plants showed a significant decline in nearly all the growth biomarkers involving root and shoot length, overall fresh and dry mass of plant in comparison to control as well as treated plants (Fig. 1). As compared to control, the root length of LT plants was decreased by 2.4 fold while G1 and G3 showed a 1.06 fold and 1.21 fold decrease in root length. On the contrary, G2 plants showed a substantial increase by 0.9 fold as compared to control plants. G1C, G2C and G3C plants displayed 1.7 fold, 1.5 fold and twofold decrease as compared to control plants, respectively. In the case of shoot length LT and G3 plants showed 1.94 fold and 1.18 fold decreases, respectively, as compared to control plants. However, G1 and G2 plants showed a rise by 0.97 fold and 0.98 fold increase, respectively as compared to control plants. G1C, G2C and G3C plants showed 1.18 fold, twofold and 1.3 fold decline in shoot length when compared to control plants.

The fresh weight of the plant was seen to be affected by LT. Maximum decline of 2.1 fold fresh weight was reported in LT plants as that of control plants. G2 plants showed a 0.98 fold rise in fresh weight as compared to control. The remaining G1, G3, G1C, G2C, and G3C plants showed a marginal decline by 1.01 fold, 1.06 fold, 1.55 fold, 1.35 fold and 1.59 fold in comparison to control plants.

**Figure 2:**
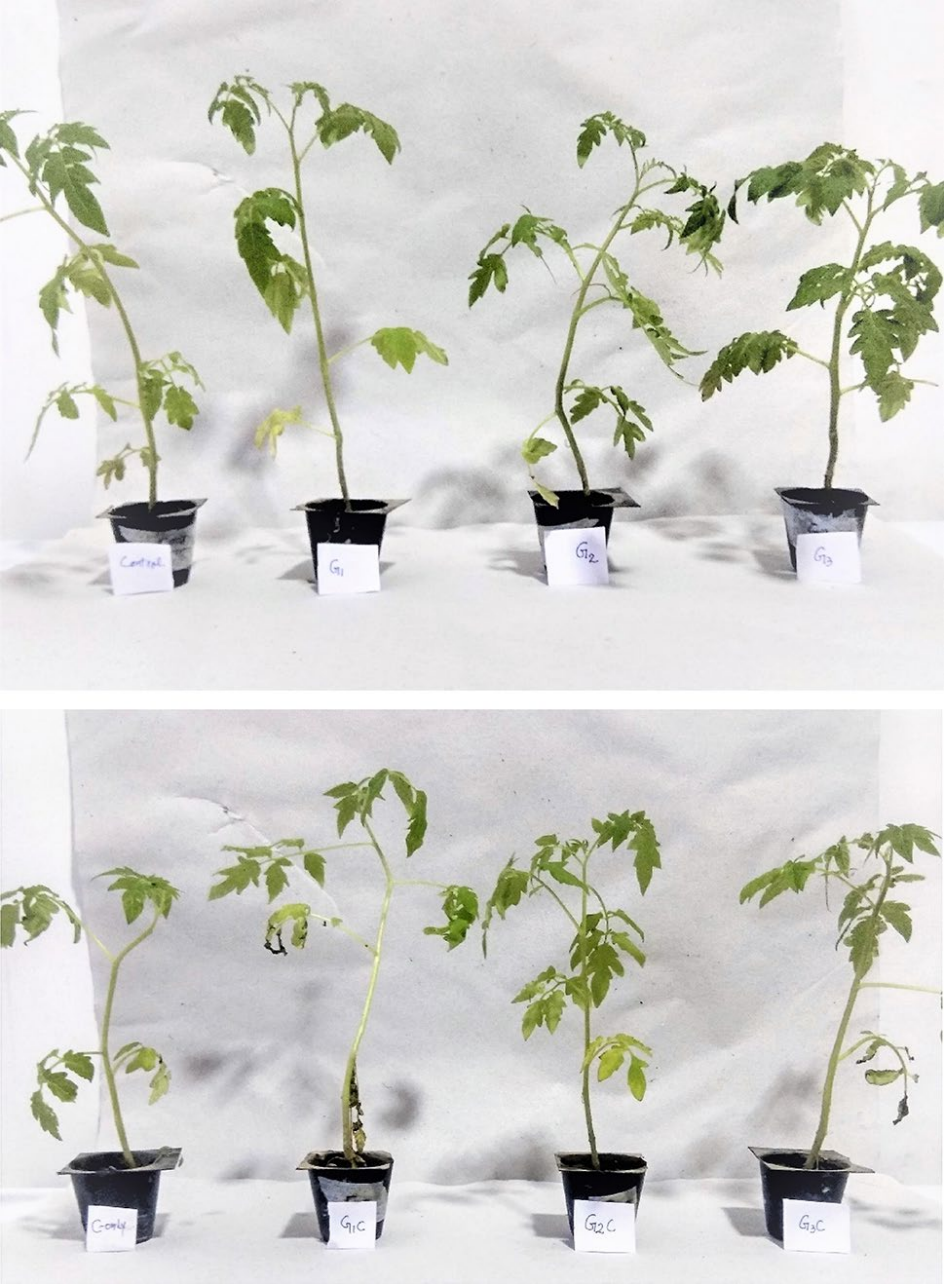
Effect of cold stress and different GSH concentration in shoot morphology. Control (Con): 25/18 °C + 0 mM GSH, G1: 25/18 °C + 0.5 mM GSH, G2: 25/18 °C + 1 mM GSH, G3: 25/18 °C + 2 mM GSH, Low temperature stress (LT/C): 10/3 °C + 0 mM GSH, G1C: 10/3 °C + 0.5 mM GSH, G2C: 10/3 °C + 1 mM GSH, G3C: 10/3 °C + 2 mM GSH.

The dry weight of plants decreased as compared to control plants. LT, G1, G2, G3, G1C, G2C, and G3C plants showed 3.25 fold, 1.15 fold, 1.04 fold, 1.4 fold, 1.67 fold, 1.26 fold and 1.7 fold decrease in dry weight as that of control plants. Least decrease in dry weight was evident by G2 concentration both in the presence and absence of LT (Fig. 2).

### Stress tolerance index

**Figure 3:**
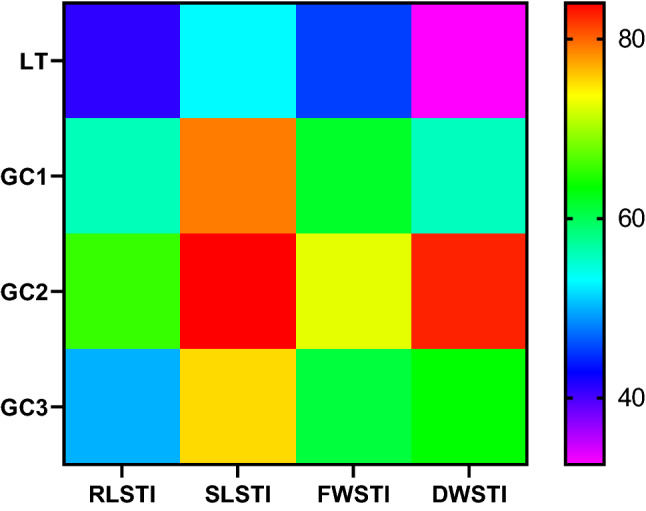
Heat map visualization of STI: Based on different stress tolerance indices (RLSTI, SLSTI, FWSTI and DWSTI) was plotted in color scale, with blue indicating least and red indicating maximum tolerance potential. Low temperature stress (LT): 10/3 °C + 0 mM GSH, G1C: 10/3 °C + 0.5 mM GSH, G2C: 10/3 °C + 1 mM GSH, G3C: 10/3 °C + 2 mM GSH.

Cold stress lowers the tolerance level of Pusa sheetal cv. of *S. lycopersicum*. However, exogenous supplementation of GSH tends to increase the tolerance level of the same. Root length stress tolerance index (RLSTI) and shoot length stress tolerance index (SLSTI) are maximally seen in GC2 plants (66% and 84% respectively) while least are seen in LT plants (40% and 53% respectively). The highest value of fresh weight (FW) and dry weight (DW) were recorded in GC2 plants (73% and 82.6%, respectively). The least value FW and DW was seen in LT plants (45.4% and 32% respectively). Marginal RLSTI (56%), SLSTI (79%), FWSTI (62%) and DWSTI (55.7%) was seen in G1C plants. G3C plants were noted to have RLSTI (50%), SLSTI (75%), FWSTI (61%) and DWSTI (63%) (Fig. 3).

### Gas exchange parameters

**Figure 4:**
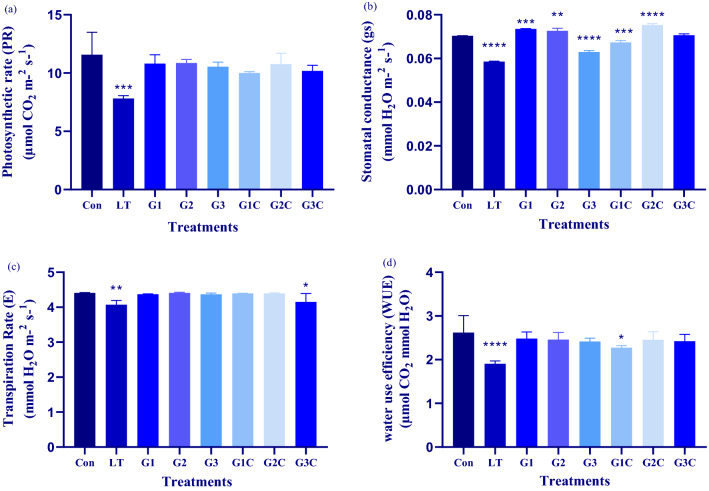
Effect of cold stress and different GSH concentrations on leaf gas exchange parameters: Forty days after sowing seedlings were used for experimentation pertaining to LT stress and exogenously applied GSH. Post 3 days recovery determination of (**a**) photosynthetic rate, (**b**) stomatal conductance, (**c**) Transpiration rate and (**d**) water use efficiency was done. The data depicts the mean and standard deviation of three replicates. Data followed by (*) determines level of significance (*p* < 0.05) as predicted by Dunnet’s multiple comparison test. Control (Con): 25/18 °C + 0 mM GSH , Low temperature stress (LT): 10/3 °C + 0 mM GSH, G1: 25/18 °C + 0.5 mM GSH, G2: 25/18 °C + 1 mM GSH, G3: 25/18 °C + 2 mM GSH, G1C: 10/3 °C + 0.5 mM GSH, G2C: 10/3 °C + 1 mM GSH, G3C: 10/3 °C + 2 mM GSH.

There were significant variations in all of the gas exchange metrics examined across all treatments. Net photosynthetic rate (PN) was noticed remarkably lesser in the case of LT plants (7.88 µmolCO_2_ m^−2^ s^−1^) as compared to control plants (11.56 µmolCO_2_ m^−2^ s^−1^). GSH only treated plants G1, G2 and G3 plants have 11.2 µmolCO_2_ m^−2^ s^−1^, 10.8 µmolCO_2_ m^−2^ s^−1^ and 10.5 µmolCO_2_ m^−2^ s^−1^ PN rate. While LT + GSH plants including G1C, G2C and G3C have 10 µmolCO_2_ m^−2^ s^−1^, 10.7 µmolCO_2_ m^−2^ s^−1^ and10.2 µmolCO_2_ m^−2^ s^−1^. Stomatal conductance (gs) was found to be significantly lower in the case of LT plants (0.058 mmol H_2_O m^−2^ s^−1^) when compared with normal plants (0.070 mmol H_2_O m^−2^ s^−^1). However, the transpiration rate (E) was found to be lower in LT as compared to control and LT + GSH plants. Water use efficiency (WUE) was found to be significantly higher in control plants followed by GSH treated plants (Fig. 4a–d).

### Correlation based analysis 2D contour plotting

**Figure 5:**
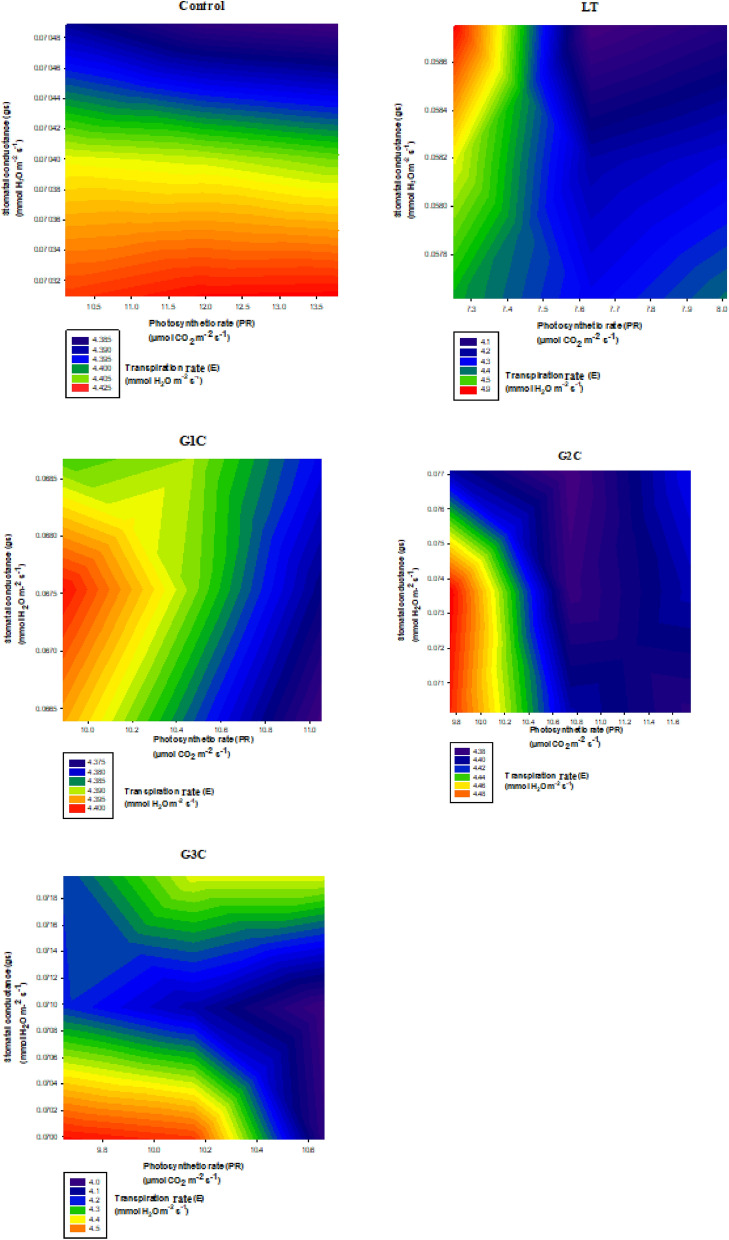
2D contour plots showing relationship between stomatal conductance, photosynthetic rate and transpiration rate under low temperature stress in accordance with varying concentration of GSH. Control (Con): 25/18 °C + 0 mM GSH, Low temperature stress (LT): 10/3 °C + 0 mM GSH, G1C: 10/3 °C + 0.5 mM GSH, G2C: 10/3 °C + 1 mM GSH, G3C: 10/3 °C + 2 mM GSH.

The 2D contour maps explain the response surface system. The oval shape of contour lines depicts the significant interaction between the variables while the insignificant type of interactions are generally shown by straight lines^22^. Here, in this analysis, the 3D form of data is visualized by a 2D pattern (Fig. 5). The distance between the contour lines represents the steepness of the slope, the lesser the distance, the more is the steepness (changing pattern of interaction), while the larger space between contour lines represents a soft slope (lesser changing pattern) while no lines show flat region (constant type of interaction). In the case of control contour plots, the lines show a normal pattern with a gentle slope and flat regions were also seen under transpiration rates of 4.4 mmol H_2_O m^−2^ s^−1^ and 4.3 mmol H_2_O m^−2^ s^−1^ respectively. Contrary to this, LT only stressed plants show sharp steepness of contour lines with prominent deviation from an oval shape to a straight line. This signifies the less interaction between stomatal conductance, photosynthetic rate and transpiration rate. The effect of G1C concentration of GSH on the plant under LT stress show plots with less steepness and with a gentle slope at 4.390 mmol H_2_O m^−2^ s^−1^ to 4.395 mmol H_2_O m^−2^ s^−1^. However, the G2C supplementation shows a very feeble level of steepness with a promising highly flat region under 4.38 mmol H_2_O m^−2^ s^−1^ transpiration rate. This further validates the high level of interaction between stomatal conductance, photosynthetic rate and transpiration rate. Also, G3C depicts a low level of steepness with minor deviation from the oval pattern, however, less interaction was seen as compared to G1C and G2C treatments. Therefore in the current case, the maximum interaction was seen under G2C treatment followed by G1C and G3C respectively as compared to that of LT only stressed plants.

### Oxidative stress

#### Lipid peroxidation Level

**Figure 6:**
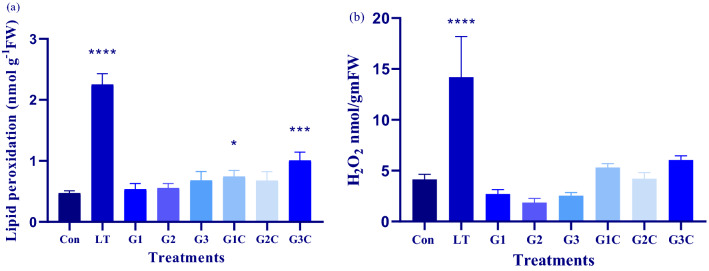
Effect of LT stress and GSH on oxidative stress parameters: Forty days after sowing seedlings were used for experimentation pertaining to LT stress and exogenously applied GSH. Post 3 days recovery determination of (**a**) lipid peroxidation (**b**) hydrogen peroxide content was carried. The data depicts the mean and standard deviation of three replicates. Data followed by (*) determines level of significance (p < 0.05) as predicted by Dunnet’s multiple comparison test. Control (Con): 25/18 °C + 0 mM GSH, Low temperature stress (LT): 10/3 °C + 0 mM GSH , G1: 25/18 °C + 0.5 mM GSH , G2: 25/18 °C + 1 mM GSH, G3: 25/18 °C + 2 mM GSH, G1C: 10/3 °C + 0.5 mM GSH, G2C: 10/3 °C + 1 mM GSH, G3C: 10/3 °C + 2 mM GSH.

To evaluate the LT stress-associated cellular membrane damage (MDA content), estimation in terms of TBAR formation was carried out. Quantification of TBAR content under LT stress (LT) and non-LT stress (Control) was performed. However, the influence of exogenous GSH on TBAR content under LT stress (G1C, G2C, G3C) and non LT stress (G1, G2, G3) conditions were also analyzed. Analysis was carried out in terms of fold change in lipid peroxidation level. A significant 4.7-fold increase in levels of TBAR content was seen in LT plants as that of the control plant (Fig. 6a). In GSH only treated plants i.e. G1, G2 and G3 level of TBAR was the same around control plants. But in LT + GSH treated plants i.e. G1C, G2C and G3C substantial decline in TBAR content by threefold, 3.3 fold and 2.2 fold was seen compared to LT only plants. The minimum TBAR content was seen under G2C treatment and the maximum was present in LT plants. Hence, GSH-treated plants were able to decrease the membrane damage under LT stress conditions.

#### H_2_O_2_ content

Reactive oxygen species (ROS) formed as a result of LT stress was assessed in this study with regard to H_2_O_2_ buildup. We assessed variation of the H_2_O_2_ level under LT stress and GSH supplementation. A substantial increase by 3.4 fold was seen in H_2_O_2_ content in LT plants as compared to control plants. A decrease in H_2_O_2_ was seen in G1, G2, and G3 treated plants by 1.5 fold, 2.3 fold, and 1.7 fold respectively in comparison with control. However, as compared to LT + GSH plants, G1C, G2C and G3C showed 2.6 fold, 3.3 fold and 2.3 fold reduction in H_2_O_2_ content (Fig. 6b). There was a significant decline of H_2_O_2_ formation in the case of LT + GSH as that of LT plants. The maximum decline was seen among G2C plants. Thereby, we conclude that GSH helps to mitigate the H_2_O_2_ accumulation in *S. lycopersicum*.

### CAT activity

**Figure 7:**
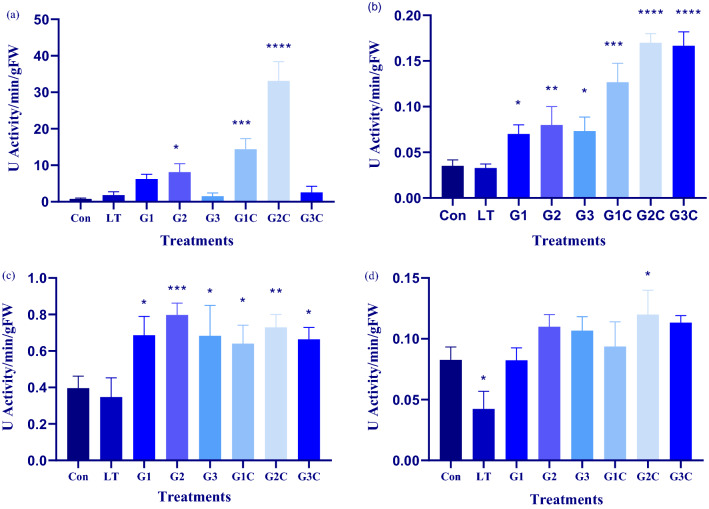
Effect of cold stress and different GSH concentrations on antioxidant activity: Forty days after sowing seedlings were used for experimentation pertaining to LT stress and exogenously applied GSH. Post 3 days recovery determination of antioxidant activities (**a**) CAT (**b**) GR (**c**) SOD (d) APX was carried. The data depicts the mean and standard deviation of three replicates. Data followed by (*) determines level of significance (*p* < 0.05) as predicted by Dunnet’s multiple comparison test. Control (Con): 25/18 °C + 0 mM GSH , Low temperature stress (LT): 10/3 °C + 0 mM GSH , G1: 25/18 °C + 0.5 mM GSH , G2: 25/18 °C + 1 mM GSH, G3: 25/18 °C + 2 mM GSH, G1C: 10/3 °C + 0.5 mM GSH, G2C: 10/3 °C + 1 mM GSH, G3C: 10/3 °C + 2 mM GSH.

The activity of CAT enzyme in the leaves of *S. lycopersicum* seedlings was affected by LT stress and GSH treatment. LT + GSH (G1C, G2C and G3C) plants and only GSH (G1, G2, and G3) resulted in a surge in CAT activity. As compared to control plants 8.8 fold, 11.5 fold and 2.14 fold increase in CAT activity was deduced in G1, G2, and G3 treated plants, respectively. In comparison to the control plant, there is a little increase in CAT activity was also seen in LT only plants (Fig. 7a). However, in LT + GSH treated plants eightfold, 18.3 fold and 1.3 fold increase in CAT activity was seen in G1C, G2C and G3C, respectively. Hence, G2 concentrations have profoundly increased the activity of CAT under normal and LT stress conditions.

### Glutathione reductase activity

In the current study, the assessment of enzymatic activity of GR that is involved in preserving a high GSH/GSSG pool, which is critical for imparting tolerance under LT stress was done. Our study demonstrates, the level of GR activity in LT stressed and control plants was the same. However, it increased 1.8 fold, 1.9 fold and 2.1 fold, respectively in GSH treated plants. GR activity increased in LT + GSH treated plants involving G1C, G2C and G3C by 3.5 fold, 4.4 fold and 4.2 fold, respectively when compared to LT only. G2C has the highest level of GR activity and LT being the least (Fig. 7b). Subsequently, GR activity increased in Pusa sheetal plants by exogenous application of GSH.

### Superoxide dismutase activity

Evaluation of SOD enzyme activity that is seen as an initial line of defense to quench superoxide-free radicles generated due to LT stress was also measured. Plants treated with GSH had higher SOD activity than control plants, but LT plants had lower SOD activity. Only GSH treated plants G1, G2 and G3 showed 1.7 fold, twofold and 1.7 fold increase, respectively as that of control. LT + GSH treated plants including G1C, G2C and G3C have 1.8 fold, 2.14 fold, and 1.9 fold increase in SOD activity in comparison to that of LT stressed plants (Fig. 7c). SOD activity was better in G2C and least in LT plants. Thus, like other antioxidants SOD activity increased in presence of GSH.

### Ascorbate peroxidase activity

LT stress is accompanied by H_2_O_2_ accumulation. APX enzyme is having H_2_O_2_ scavenging role. The activity of APX increased by GSH treatment to plants. G1, G2 and G3 showed onefold, 1.3 fold and 1.1 fold increase in activity of APX as compared to control. But in LT + GSH plants 2.2 fold, 2.8 fold and 2.6 fold increase was seen in G1C, G2C and G3C, respectively in contrast to that of LT only. So, G2C has maximum AXP activity (Fig. 7d). Henceforth, LT stress leads to a decline in APX activity while GSH application increases its activity in LT as well as in normal conditions.

## Discussion

**Figure 8:**
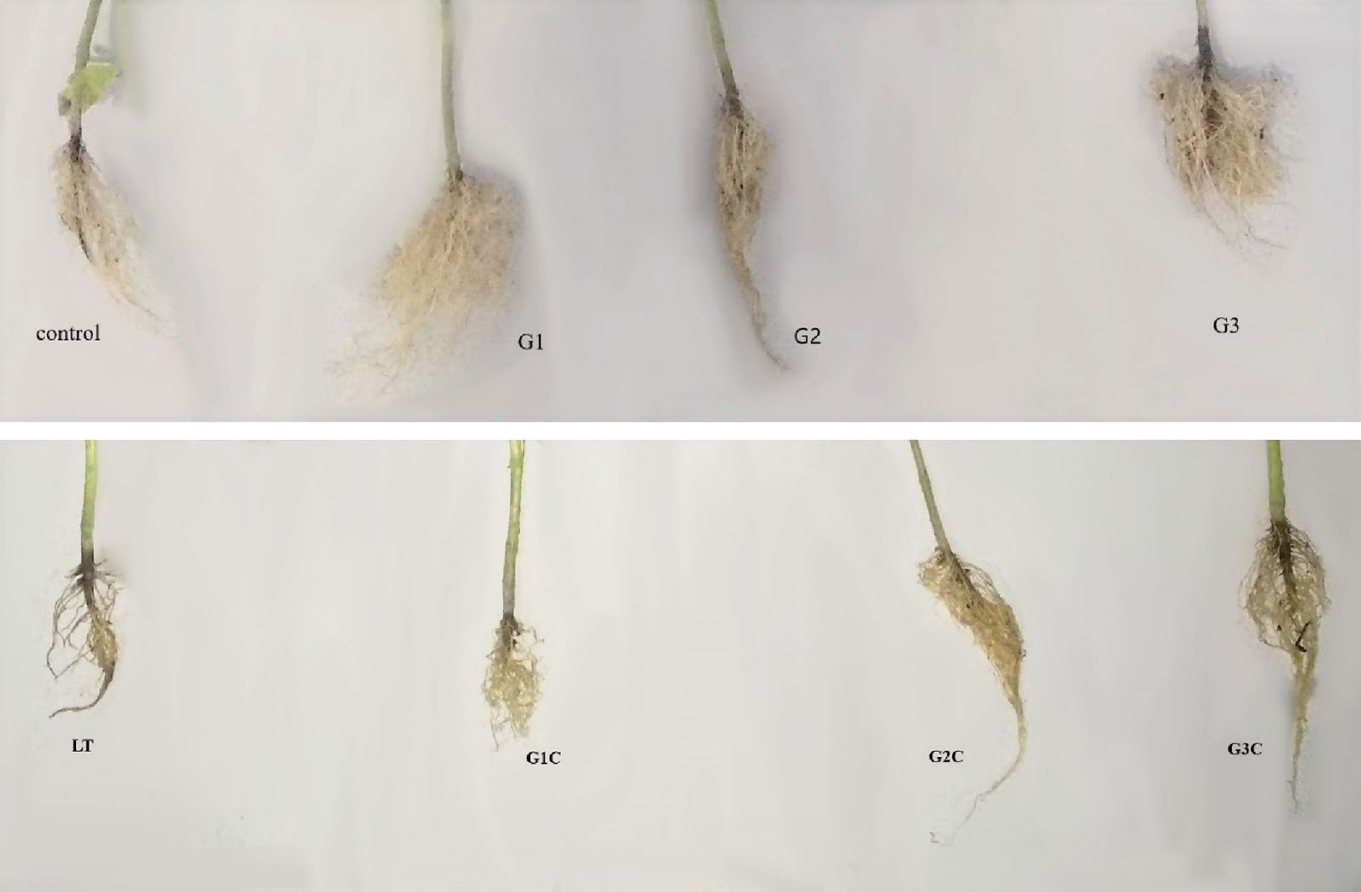
Changes pertaining to low temperature stress and different GSH concentration in root morphology. Control (Con): 25/18 °C + 0 mM GSH, Low temperature stress (LT): 10/3 °C + 0 mM GSH, G1: 25/18 °C + 0.5 mM GSH, G2: 25/18 °C + 1 mM GSH, G3: 25/18 °C + 2 mM GSH, G1C: 10/3 °C + 0.5 mM GSH, G2C: 10/3 °C + 1 mM GSH, G3C: 10/3 °C + 2 mM GSH.

Under abiotic conditions, GSH is responsible for controlling a variety of physiological responses in plants but its role in mitigating cold stress tolerance in *S. lycopersicum* (Pusa sheetal cv.) has not yet been studied so far. In the present study, GSH was found to combat cold stress in *S. lycopersicum* by enhancing antioxidant machinery, biochemical traits, photosynthetic parameters and growth characteristics. LT stress has a significant effect on plant growth and developmental processes. Plant progress is influenced by LT. It initiates cascades of physiological, biochemical and morphological changes that limit the productivity of plants^23^. In the present study, LT caused a decrease in seedling growth with evident chilling injury as revealed by reduced SL, RL, FW and DW. It is in accordance with the previous as well as recent reports involving wheat^24^, barley^25^ and rice^26^. There could be many reasons for the retarded growth of plants under LT stress like ROS production, improper nutrient uptake and osmotic imbalance^27^. However, GSH treated LT (GSH + LT) stressed seedlings were able to combat the detrimental effects of LT with better SL, RL, FW and DW (Fig. 8). Moreover, it is already reported that elevated levels of constitutive GSH increase cell division of the meristematic zone of the root region which leads to root elongation^28^. GSH treatment i:e; GSH only and LT + GSH enhanced root growth higher than control plants and LT-only plants, respectively. However, the progressive effect of exogenous GSH in controlling growth, development and yield under abiotic stress has been reported in *Arabidopsis* mung bean and soybean^11,29,30^. This study also indicates that GSH treatment has protective roles in lessening the toxic effects of LT on the growth and development of *S. lycopersicum*. It has been already reported that osmoregulators and antioxidants have a defined role in imparting abiotic stress tolerance like salinity stress^31^ Moreover, the absorptive surface area of root (Fig. 8) were considerably elevated in GSH-supplied plants than in control and LT plants. This kind of scenario has also been described in maize under different abiotic stresses by Pei et al.^32^. Different root growth scenario was also evaluated in tomato cultivars under salt stress reported by Zaki et al.^33^.

Photosynthesis is one of the principal physiological processes of plant systems that depend on various elements like light, fixation of CO_2_ and other abiotic factors including temperature^34^. LT has been found to lower the CO_2_ assimilation thereby reducing the rate of photosynthesis^35^. In the current experiment LT only plants have the least levels of photosynthesis as compared to the control. Exogenously applied GSH under normal or LT temperature tends to increase the rate of photosynthesis when compared to that of control and LT only respectively. This could be due to the thioredoxin property of GSH due which it controls the different enzymes of photosynthesis. However, it is also suggested that the reduced state of GSH in predominance protects the activity of the main enzymes of photosynthesis^36^. GSH probably protects the active sites from inhibitor binding that would halt the process of photosynthesis^37^. The transpiration rate, stomatal conductance and water usage efficiency increased in GSH treated plants as compared to LT only and the levels were comparable to that of control plants. This is in accordance with the photosynthetic performance shown by GSH increased transpiration rate (E), net photosynthetic rate (PN), and stomatal conductance (gs) abiotic stress in maize genotypes^38^. Furthermore, the membrane-stabilizing effect of GSH application could be a main protective mechanism for GSH-induced LT stress relief. GSH triggers signaling that regulates cellular redox state and protects fauna from abiotic stress while also maintaining cell membrane integrity^39,40^.

The ability of stomata to regulate their aperture to minimize water loss while maintaining CO_2_ uptake is the intricate mechanism that favors the plant to persist under unfavorable conditions^41^*.* Stomatal movements (opening and closure) control the CO_2_ usage and water loss via evaporation in response to environmental factors^42^. Decreased stomatal conductance results decline in the rate of photosynthesis by limiting the uptake of CO_2,_ while high stomatal conductance favors high photosynthesis output^43,44^. It is due to high stomatal conductance which causes the high rate of CO_2_ uptake thereby increasing photosynthesis^42^. The cold stress causes the closure of stomata. Furthermore, it has been reported that abscisic acid (ABA) buildup occurs during LT stress in plants. The ABA has been reported to enhance stomatal closure. GSH depletion is also caused by ABA in guard cells. In addition, the GSH mutant plants show enhanced ABA-mediated stomatal closure^45^. But as per early reports, the GSH redox pool of cells has a promising effect on ABA signaling in plants^46^. The 2D contour maps are suggestive of significant interaction between variables while an insignificant type of interaction is shown by straight lines^22^. To demonstrate the type and the level of interaction between photosynthesis, the conductance of stomata, and transpiration rate 2D contour plots were taken into account. Contour plotting reveals that the interaction between these three parameters has an important role in combating cold stress tolerance in *S. lycopersicum* plants. From these plots, it is quite evident that G2C concentration of GSH favors this kind of interaction at maximum. Therefore, this kind of interaction seems to be an important factor that determines the LT stress alleviating capacity of the plants. More is the interaction between stomatal conductance, photosynthetic rate and transpiration rate more will be LT stress bearing tendency of *S. lycopersicum* plants. Our study also suggest that G2C favors this kind of interaction apart from boosting the antioxidant machinery of *S. lycopersicum* plants. This could be of suggestive that G2C concentration halts ABA mediated stomatal closure to much higher extend than G1C and G3C. As G2C concentration is almost having same levels or minute diminished levels of photosynthetic rate, stomatal conductance, and transpiration rate respectively in comparison with control. Meanwhile, G2C concentration helps to maintain its integrity by regulating stomatal conductance that which effect photosynthetic rate and transpiration rate. Hence, G2C may have promising role to enhance the GSH redox and inhibiting ABA mediating signaling in *S. lycopersicum* under cold stress conditions.

The stress tolerance index (STI) is a valuable way of defining the stress tolerance potential. The SL, RL, FW and DW are important parameters for the classification of tolerance^47^. STI suggests a tolerance mechanism that allows plants to retain development even in the presence of abiotic factors such as hazardous metal levels^48^. In this study, maximum stress tolerance level was shown by G2C concentrations of GSH under LT stress followed by G1C and G3C. LT only plants decipher the least levels of STI. Consequently, GSH helps to increase STI of *S. lycopersicum* under LT stress. These findings correlate with data suggesting that the improved GSH level results in stress tolerance as seen in *Arabidopsis*^29^. In addition to imparting stress tolerance by GSH treatment, a rise in the components of the electron transport chain can be altered by GR activity. The involvement of GR is in preserving the decreased GSH level and control the cellular ROS scavenging phenomenon under stressful conditions^49^.

GSH being regarded as the main cellular antioxidant that acts as determining factor of the cellular redox state by regulating various redox signaling by reacting with ROS that is produced in response to various stresses thereby acting as a major scavenger^50,51^. Unfavorable environmental causes overproduction of ROS that leads to considerable changes in cellular lipid membrane causing peroxidation of lipids (shown by increased MDA)^52^. It was reported by Nahar et al.^13^ that supplementation of GSH to mung bean seedlings improved tolerance level to high temperatures. Furthermore, Pei et al.^32^ also reported that antioxidant activities involving SOD, CAT, GR and APX were reduced under abiotic treatment while the application of GSH enhanced their activity. Our results are in accordance with these findings that in imparting LT stress tolerance GSH is having important role via boosting the antioxidant capability of *S. lycopersicum*. Also, the level of membrane damage and H_2_O_2_ content was seen least in plants treated with GSH. Hence, these results suggest that the decline in oxidative stress due to cold stress was ameliorated by GSH treatment which could be due to enhanced antioxidant capability thereby increasing stress tolerance observed in GSH-treated plants. Moreover, the reduced form of GSH directly detoxifies ROS and controls the activities pertaining to GSH dependent ROS and MG detoxifying enzymes^9,53^. In addition, it is an important part of the ascorbate -glutathione (AsA-GSH) cycle that regulates levels of H_2_O_2_ in plant cells. Plants generally maintain high levels of GSH/GSSG ratio. GSH reacts with ROS species and gets transformed to GSSG causing a decline in GSH/GSSG ratio that results in oxidative stress^10,11^. So, the exogenous application of GSH keeps the GSH/GSSG pool in check to decrease the level of membrane damage apart from increasing antioxidant machinery. Among different GSH concentrations, G2 showed more promising antioxidant activities and lesser oxidative stress build-up in *S. lycopersicum* under LT stress. At higher concentrations, G3 response were not good as compared to G2 concentration. Reason could be that G2 concentration suit its physiological level as GSH treatment has been reported to increase the levels of ABA and jasmonic acid in plants. When plants being exposed to high concentration GSH causes evident rise in the endogenous level of ABA and Jasmonates that might have led to reduced growth and increase in LP even under normal conditions^29^.

## Conclusion

In conclusion, exogenous supplementation alleviates the LT stress in Pusa sheetal cv. of *S. lycopersicum* plant. G2 concentration has immense potential to combat LT stress. Several means by which GSH encounters LT stress could be concluded in the following points.

1. Maintaining osmotic equilibrium and membrane integrity.
2. The activity of antioxidant enzymes including CAT, GR, APX, and SOD has enhanced.
3. Advanced growth and development by regulating SL, RL, FW and DW.
4. GSH aided in the increased rate of photosynthesis.
5. Improved the efficacy of gaseous exchange parameters like stomatal conductance, transpiration level and water usage efficiency.
6. Progress in stress tolerance indices as compared to LT stressed plants.
7. LT stress-bearing tendency depends on a favorable kind of interaction between stomatal conductance, photosynthetic rate and transpiration rate as seen under G2C treatment.
8. Under LT stress circumstances, exogenous GSH most prominently G2C concentration may have a potential role in increasing GSH redox and suppressing ABA-mediated signaling in Pusa sheetal cv. of *S. lycopersicum*.

Our results provide insights on the role of GSH in combating LT stress and could be a possible approach to enhance LT stress resistance in Pusa sheetal cv. of *S. lycopersicum.*

## Materials and methods

The Indian Agriculture Research Institute (IARI) in New Delhi, India, provided seeds of the Pusa Sheetal cv. of tomato. Surface sterilization of seeds with 2% sodium hypochlorite and then washed with sterile deionized water. In a growth chamber, these sterilized seeds were planted in a container containing soil made up of compost and peat (1/4, v/v) mixed with sand (3:1, v/v). 40 days after sowing (DAS), some plants were subjected to LT stress (10°/3 °C) day/night temperature (LT) for 24 h in a growth chamber rest kept at normal temperature 25/18 °C (control). While some plants were given folair GSH treatment of variable concentrations involving G1 (0.5 mM), G2 (1 mM) and G3 (2 mM) in prophylactic, preemptive and curative dosage dependent manner with LT (LT + GSH) and without LT (G). The LT stressed plants including LT + GSH and LT after 24 h were sustained under ambient conditions of day/night using 700 µmol m^−2^ s^−1^ photosynthetically active radiations, normal day/night temperature of 25/17 ± 3 °C and relative humidity 75% in the growth chamber. Sampling was done after 3 days recovery period.

### Growth data

#### Root length and shoot length

The root-shoot length specifies length of plant arise from the root tip to most growing tip of the central axis. Plants were uprooted carefully, washed and were retained on moist filter papers to avoid desiccation. With the aid of a measuring scale in cm, the root and shoot lengths were measured and recorded^54^.

#### Dry weight and fresh weight

Plants were uprooted cautiously, followed by proper washing to remove soil and weighed. Fresh weight will be deduced using balance. Plant dry mass was calculated after drying them at 80 °C in a hot air oven until constant weight is attained^55^.

### Determination of gas exchange parameters

Wholly expanded top most leaves of plants were analyzed under infrared gas analyzer (IRGA, Model LI6400XT, LI-COR Lincoln, Nebraska, USA) to determined Gas exchange parameters. The experiment was carried out between 11.00 and 12.00 h at light-saturating intensity, 2 cm^2^ of leaf area, block temperature (25 °C), CO_2_ flow controller (300 µmol s^−1^) and PAR (1600 μmol photons m^−2^ s ^−1^). Before proceeding the experiment calibration of IRGA was done that includes zeroing replacement of drierite and soda lime. The healthy third leaf from apex was taken into account for recording leaf gaseous exchange attributes like transpiration rates (E) (mmol H_2_O m^−2^ s^−1^), stomatal conductance (gs) (mmol H_2_O m^−2^ s^−1^), photosynthetic rate (PN) (µmol CO_2_ m^−2^ s^−1^) and water use efficiency (WUE). (Relationship between photosynthesis and transpiration).

### Stress tolerance index

The tolerance indices for diverse growth factors were calculated using protocol followed by Amin H et al.^47^.

### Oxidative stress

#### Lipid peroxidation level

Analysis involving peroxidation of lipids in the leaf sample of plant was carried in corresponding to thiobarbituric acid content (TBARS). Protocol suggested by Dhindsa et al.^56^ was followed. Samples were grounded in solution comprising of thiobarbituric acid (TBA 0.25%) prepared in trichloroacetic acid (TCA 10%) followed by heating them at 95 °C then cooled on ice and centrifuged for 10 min at 10,000 g. Subsequently 4 ml solution of TCA (20%) containing TBA (0.5%) was supplemented to 1 ml of supernatant. At 532 nm, the absorbance was measured. By subtracting the absorbance value of a comparable sample at 600 nm, the unspecific turbidity was adjusted. Using the extinction coefficient (155 mM^-1^ cm^-1^) the TBARS content was calculated.

#### H_2_O_2_ content determination

The estimation of H_2_O_2_ was determined suggested by Okuda et al.^57^. A fresh leaf sample was powdered in cooled perchloric acid (200 mM) for 10 min and then centrifuged at 1300 g. The supernatant containing perchloric acid was neutralized using 4 M potassium hydroxide (KOH). Centrifugation was used to remove the residual insoluble potassium perchlorate. The total amount of 1.5 ml contains eluate (1 ml), 3-methyl-2-benzothiazoline hydrazine (80 µl), 3-(dimethylamino) benzoic acid (12.5 mM, 400 µl) in phosphate buffer (0.375 M, pH 6.5) and 20 µl (0.25 unit) of peroxidase was prepared. At 590 nm, the increase in absorbance was recorded.

#### Catalase assay

The protocol of Aebi (1984) was used to deduce the CAT activity^58^. Fresh leaf sample grounded in extraction buffer comprising of Na-phosphate (0.5 M, pH 7.3), EDTA (3 mM), Triton X 100 (1% v/v) and PVP (1% w/v). Then centrifugation at 13280 g for 25 min at 4 °C was done. The final reaction comprises of 2 ml of Na-phosphate buffer (pH 7.3, 0.5 M,) 0.1 ml of enzyme extract, 0.1 ml of EDTA (3 mM) and 0.1 ml of H_2_O_2_ (3 mM) for 5 min. observing the depletion of H_2_O_2_ in accordance with a drop in absorbance at 240 nm was used to measure CAT activity in the supernatant. For calculation purpose, 0.036 mM^-1^ cm^-1^ was considered as coefficient of absorbance. The quantity required to decompose 1 µmol of H_2_O_2_ per minute determines unit activity of enzyme.

#### Glutathione reductase assay

This activity was determined using Anderson^59^. The total reaction mixture (1 ml) have oxidized glutathione (0.02 mM, GSSG) and NADPH (0.2 mM) in a potassium phosphate buffer (0.1 M, pH 7.2). After adding enzyme extract (0.2 ml) to the mix, the process began. The activity was deciphered by fall in absorbance at 25 °C for 340 nm for 3 min. The conversion of 1 µmol of GSSG min^-1^ at 25 °C gives unit enzyme activity.

#### Superoxide dismutase

The Dhindsa et al.^56^ method was followed to perform SOD assay relay on the capability of SOD to halt of formation of nitroblue tetrazolium (NBT) by photochemical reduction. The total reaction mixture consisting of 1.5 ml sodium phosphate buffer (0.1 M, pH 7.5) and PVP (1% w/v) L-methionine (13 mM), enzyme extract (0.1 ml) with same amounts of NBT solution (2.25 mM), riboflavin (60 μM), Na_2_CO_3_ (1 M), EDTA (3 mM) and double-distilled water (1.0 ml, DDW). Samples were then irradiated at 28 °C under 15 W fluorescent lamp. At 560 nm the absorbance of the irradiated samples was compared to the non-irradiated samples. The quantity of enzyme extract equivalent to 50% reduction (Percent inhibition of colour) of NTB was taken as enzyme activity (single unit).

#### Ascorbate peroxidase activity (APX).

APX activity was carried out by the protocol of Nakano and Asada^60^. Centrifugation of Fresh leaf sample grounded in potassium-phosphate extraction buffer (0.1 M, pH 7, 5cm^3^), Triton X 100 (1%), EDTA (3 mM), PVP (1%) was done at 4 °C for 10 min at 7800 g. The drop in ascorbate absorbance at 290 nm was used to calculate APX activity in the supernatant. Total reaction volume contains buffer (1 cm^3^) contained ascorbate (0.5 mM), EDTA (0.1 mM), H_2_O_2_ (0.1 mM) and extract of enzyme (0.05 cm^3^). The reaction was proceeded at 25 °C for 5 min. By using coefficient of absorbance 2.8 mM^-1^ cm ^-1^ APX activity was calculated. The amount essential to decompose 1 μmol of ascorbate per minute determines one unit of enzyme.

### Statistical analysis

Each experiment included the set three plants for each treatment. Graph-pad prism 8 software for Windows was used for statistical analysis. The analysis of variance (ANOVA) test was used to evaluate the significant differences between parameters. The value of *p* ≤ 0.05 was used to compare means. The data depicts the mean and standard deviation of three replicates. Data followed by (_*_) determines level of significance (*p* < 0.05) as predicted by Dunnet’s multiple comparison test. 2D contour plots were plotted using Sigma 14. 5 Software package.

### Ethical approval

The seeds utilized in this study were obtained from the Indian Agriculture Research Institute (IARI) in New Delhi, which governs the seed manufacturing and processing. This study complies with relevant institutional, national, and international guidelines and legislation.

## Data Availability

The datasets analyzed during the current study are not publicly available as it is meant to be published as Meta data sharing may publicise long term aim of this research however, data can be available from the corresponding author on reasonable request.

## References

[1] Bulgari R, Franzoni G, Ferrante A (2019). Biostimulants application in horticultural crops under abiotic stress conditions. Agronomy.

[2] Mickelbart MV, Hasegawa PM, Bailey-Serres J (2015). Genetic mechanisms of abiotic stress tolerance that translate to crop yield stability. Nat. Rev. Genet..

[3] Ruelland E, Vaultier M-N, Zachowski A, Hurry V (2009). Cold signalling and cold acclimation in plants. Adv. Bot. Res..

[4] Jan N, Andrabi KI (2009). Cold resistance in plants: A mystery unresolved. Electron. J. Biotechnol..

[5] Waraich EA, Ahmad R, Halim A, Aziz T (2012). Alleviation of temperature stress by nutrient management in crop plants: A review. J. soil Sci. Plant Nutr..

[6] Hajihashemi S, Noedoost F, Geuns J, Djalovic I, Siddique KHM (2018). Effect of cold stress on photosynthetic traits, carbohydrates, morphology, and anatomy in nine cultivars of Stevia rebaudiana. Front. Plant Sci..

[7] Decros G (2019). Get the balance right: ROS homeostasis and redox signalling in fruit. Front. Plant Sci..

[8] Chang J (2020). CBF-responsive pathway and phytohormones are involved in melatonin-improved photosynthesis and redox homeostasis under aerial cold stress in watermelon. Acta Physiol. Plant..

[9] Mostofa MG, Saegusa D, Fujita M, Tran L-SP (2015). Hydrogen sulfide regulates salt tolerance in rice by maintaining Na+/K+ balance, mineral homeostasis and oxidative metabolism under excessive salt stress. Front. Plant Sci..

[10] Mostofa MG, Seraj ZI, Fujita M (2015). Interactive effects of nitric oxide and glutathione in mitigating copper toxicity of rice (Oryza sativa L.) seedlings. Plant Signal. Behav..

[11] Nahar K, Hasanuzzaman M, Alam MM, Fujita M (2015). Roles of exogenous glutathione in antioxidant defense system and methylglyoxal detoxification during salt stress in mung bean. Biol. Plant..

[12] Ge, C. *et al.* Effects of glutathione on the ripening quality of strawberry fruits. in *AIP Conference Proceedings* vol. 2079 20013 (AIP Publishing LLC, 2019).

[13] Nahar K, Hasanuzzaman M, Alam MM, Fujita M (2015). Glutathione-induced drought stress tolerance in mung bean: coordinated roles of the antioxidant defence and methylglyoxal detoxification systems. AoB Plants.

[14] Wu, J. C., Sun, S. H., Ke, Y. T., Xie, C. P. & Chen, F. X. Effects of glutathione on chloroplast membrane fluidity and the glutathione circulation system in young loquat fruits under low temperature stress. In *III International Symposium on Loquat 887* 221–225 (2010).

[15] Cuvi MJA, Vicente AR, Concellón A, Chaves AR (2011). Changes in red pepper antioxidants as affected by UV-C treatments and storage at chilling temperatures. LWT-Food Sci. Technol..

[16] Jin X, Yang X, Islam E, Liu D, Mahmood Q (2008). Effects of cadmium on ultrastructure and antioxidative defense system in hyperaccumulator and non-hyperaccumulator ecotypes of Sedum alfredii Hance. J. Hazard. Mater..

[17] Zhang F (2011). The ICE-CBF-COR pathway in cold acclimation and AFPs in plants. Middle East J. Sci. Res..

[18] Raiola A, Rigano MM, Calafiore R, Frusciante L, Barone A (2014). Enhancing the health-promoting effects of tomato fruit for biofortified food. Mediators Inflamm..

[19] Wolf S, Yakir D, Stevens MA, Rudich J (1986). Cold temperature tolerance of wild tomato species. J. Am.
Soc. Hort. Sci..

[20] Foolad MR, Lin GY (2001). Relationship between cold tolerance during seed germination and vegetative growth in tomato: Analysis of response and correlated response to selection. J. Am. Soc. Hortic. Sci..

[21] Tiwari RN, Choudhury B, Pachauri DC (1990). ’Pusa Sheetal’can set fruit at low temperature. Indian Hortic..

[22] Myers, R. H., Montgomery, D. C. & Anderson-Cook, C. M. *Response surface methodology: process and product optimization using designed experiments*. (John Wiley & Sons, 2016).

[23] Foolad MR, Lin GY (2000). Relationship between cold tolerance during seed germination and vegetative growth in tomato: Germplasm evaluation. J. Am. Soc. Hortic. Sci..

[24] Boutraa T, Akhkha A, Al-Shoaibi AA, Alhejeli AM (2010). Effect of water stress on growth and water use efficiency (WUE) of some wheat cultivars (Triticum durum) grown in Saudi Arabia. J. Taibah Univ. Sci..

[25] Hellal FA (2018). Influence of PEG induced drought stress on molecular and biochemical constituents and seedling growth of Egyptian barley cultivars. J. Genet. Eng. Biotechnol..

[26] Sohag AAM (2020). Exogenous glutathione-mediated drought stress tolerance in Rice (Oryza sativa L.) is associated with lower oxidative damage and favorable ionic homeostasis. Iran. J. Sci. Technol. Trans. A Sci..

[27] Forni C, Duca D, Glick BR (2017). Mechanisms of plant response to salt and drought stress and their alteration by rhizobacteria. Plant Soil.

[28] Vernoux T (2000). The Root Meristemless1/Cadmium Sensitive2 gene defines a glutathione-dependent pathway involved in initiation and maintenance of cell division during postembryonic root development. Plant Cell.

[29] Cheng M-C (2015). Increased glutathione contributes to stress tolerance and global translational changes in Arabidopsis. Plant J..

[30] Akram S (2017). Exogenous glutathione modulates salinity tolerance of soybean [Glycine max (L.) Merrill] at reproductive stage. J. Plant Growth Regul..

[31] Zaki HEM, Radwan KSA (2022). The use of osmoregulators and antioxidants to mitigate the adverse impacts of salinity stress in diploid and tetraploid potato genotypes (Solanum spp.). Chem. Biol. Technol. Agric..

[32] Pei L (2019). Role of exogenous glutathione in alleviating abiotic stress in maize (Zea mays L.). J. Plant Growth Regul..

[33] Zaki HEM, Yokoi S (2016). A comparative in vitro study of salt tolerance in cultivated tomato and related wild species. Plant Biotechnol..

[34] Ribeiro RV, Machado EC, de Oliveira RF (2006). Temperature response of photosynthesis and its interaction with light intensity in sweet orange leaf discs under non-photorespiratory condition. Ciência e Agrotecnologia.

[35] Riva-Roveda L, Escale B, Giauffret C, Périlleux C (2016). Maize plants can enter a standby mode to cope with chilling stress. BMC Plant Biol..

[36] Schürmann P, Jacquot J-P (2000). Plant thioredoxin systems revisited. Annu. Rev. Plant Biol..

[37] Pietrini F, Iannelli MA, Pasqualini S, Massacci A (2003). Interaction of cadmium with glutathione and photosynthesis in developing leaves and chloroplasts of Phragmites australis (Cav.) Trin. ex Steudel. Plant Physiol..

[38] Wang F, Chen F, Cai Y, Zhang G, Wu F (2011). Modulation of exogenous glutathione in ultrastructure and photosynthetic performance against Cd stress in the two barley genotypes differing in Cd tolerance. Biol. Trace Elem. Res..

[39] Noctor G, Foyer CH (1998). Ascorbate and glutathione: Keeping active oxygen under control. Ann. Rev. Plant Physiol. Plant Mol. Biol..

[40] Kosower NS, Kosower EM (1978). The glutathione status of cells. Intl. Rev. Cytol..

[41] Hetherington AM, Woodward FI (2003). The role of stomata in sensing and driving environmental change. Nature.

[42] Willmer, C. & Fricker, M. Stomatal responses to environmental factors. In *Stomata* 126–191 (Springer, 1996).

[43] Farquhar GD, Sharkey TD (1982). Stomatal conductance and photosynthesis. Ann. Rev. Plant Physiol..

[44] Lawson T, Blatt MR (2014). Stomatal size, speed, and responsiveness impact on photosynthesis and water use efficiency. Plant Physiol..

[45] Okuma E (2011). Negative regulation of abscisic acid-induced stomatal closure by glutathione in Arabidopsis. J. Plant Physiol..

[46] Koramutla MK, Negi M, Ayele BT (2021). Roles of glutathione in mediating abscisic acid signaling and its regulation of seed dormancy and drought tolerance. Genes (Basel).

[47] Amin H, Arain BA, Amin F, Surhio MA (2014). Analysis of growth response and tolerance index of Glycine max (L.) Merr. under hexavalent chromium stress. Adv. Life Sci..

[48] Clemens S (2006). Toxic metal accumulation, responses to exposure and mechanisms of tolerance in plants. Biochimie.

[49] Gill SS (2013). Glutathione and glutathione reductase: A boon in disguise for plant abiotic stress defense operations. Plant Physiol. Biochem..

[50] Rahman I, Kode A, Biswas SK (2006). Assay for quantitative determination of glutathione and glutathione disulfide levels using enzymatic recycling method. Nat. Protoc..

[51] Hasanuzzaman M, Nahar K, Anee TI, Fujita M (2017). Glutathione in plants: biosynthesis and physiological role in environmental stress tolerance. Physiol. Mol. Biol. Plants.

[52] Gill SS, Tuteja N (2010). Reactive oxygen species and antioxidant machinery in abiotic stress tolerance in crop plants. Plant Physiol. Biochem..

[53] Ramírez L, Bartoli CG, Lamattina L (2013). Glutathione and ascorbic acid protect Arabidopsis plants against detrimental effects of iron deficiency. J. Exp. Bot..

[54] Muneer S, Ahmad J, Bashir H, Moiz S, Qureshi MI (2011). Studies to reveal importance of Fe for Cd tolerance in Brassica juncea. Int. J. Appl. Biotech. Biochem..

[55] Asgher M (2018). Ethylene supplementation increases PSII efficiency and alleviates chromium-inhibited photosynthesis through increased nitrogen and sulfur assimilation in mustard. J. Plant Growth Regul..

[56] Dhindsa RS, Plumb-Dhindsa P, Thorpe TA (1981). Leaf senescence: correlated with increased levels of membrane permeability and lipid peroxidation, and decreased levels of superoxide dismutase and catalase. J. Exp. Bot..

[57] Okuda T, Matsuda Y, Yamanaka A, Sagisaka S (1991). Abrupt increase in the level of hydrogen peroxide in leaves of winter wheat is caused by cold treatment. Plant Physiol..

[58] Aebi H (1984). [13] catalase in vitro. Methods Enzymol..

[59] Anderson ME (1985). [70] Determination of glutathione and glutathione disulfide in biological samples. Methods Enzymol..

[60] Nakano Y, Asada K (1981). Hydrogen peroxide is scavenged by ascorbate-specific peroxidase in spinach chloroplasts. Plant cell Physiol..

